# Sticky or Not: Adhesion by Architectural Design

**DOI:** 10.1021/acscentsci.3c00104

**Published:** 2023-02-03

**Authors:** Sajjad Dadashi-Silab, Erin E. Stache

**Affiliations:** Department of Chemistry and Chemical Biology, Cornell University, Ithaca, New York 14853, United States

That sticky residue left behind when a piece of electrical tape is removed—no more! In this issue of *ACS Central Science*, Dobrynin, Sheiko, and co-workers describe novel pressure-sensitive adhesives (PSAs) through the architectural engineering of bottlebrush polymers.^1^ These new adhesives are additive-free, meaning they do not leave behind a residue after debonding, typical of many commercial pressure-sensitive adhesives.

Structural adhesives, like a 5 min epoxy, form a permanent bond through a hardening process of liquid resins, often irreversibly.^2^ In contrast, the mechanism for PSA bonding involves no physical change or chemical reaction but instead adhering to any number of distinct surfaces upon contact and applying pressure. These materials are used in various commercial applications like tapes, labels, stickers, and biomedical devices. The PSA design requires imparting viscous behavior and softness for efficient bonding and elasticity while simultaneously requiring strength to withstand rupture and provide clean/easy removability upon debonding. Therefore, the design of PSAs demands a delicate balance to meet these seemingly conflicting properties.

**Figure 1:**
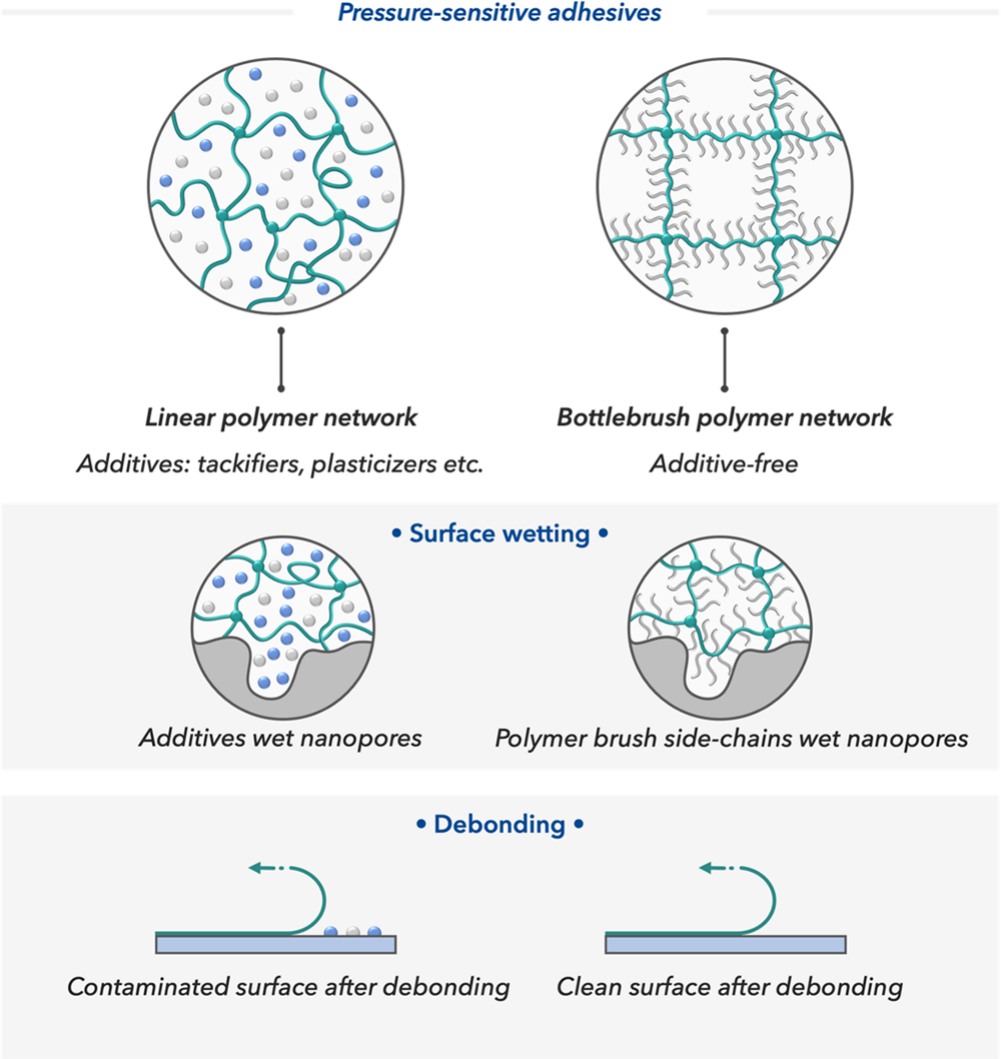
Comparison of commercial pressure-sensitive adhesives (with additives) and bottlebrush polymers (additive-free). Design principles, mechanism of action, and differences in debonding.

Pressure-sensitive adhesives are typically composed of polymeric networks that often contain small molecule additives such as plasticizers or tackifiers to tune the viscoelastic properties of the polymers (Figure 1).^3^ While the polymeric network entails stiffness and elastic properties, small-molecule additives are used to dilute the chain entanglements thus providing high surface wetting for better adhesion. The dual-ended polymer strands in a cross-linked network have limited degrees of freedom and offer limited penetration into microscopic pores, thus requiring various additives to facilitate surface wetting. However, using small-molecule additives poses further challenges as they can leach out and cause surface contamination upon debonding (sticky residue).

Through clever architectural engineering of bottlebrush polymers, Sheiko, Dobrynin, and co-workers have developed adhesives that form strong polymer networks while affording high surface wettability. Bottlebrush polymers are cylindrical macromolecules with a high density of polymeric side chains grafted in each repeat unit along the backbone of the polymer.^4^ Densely grafted side chains straighten the polymer backbone and dilute the chain entanglement, ultimately affording more elastomeric materials with distinct physical properties than their linear counterparts. In a bottlebrush polymer, highly grafted side chains dilute the mass of entanglement strands to make solvent-free, supersoft elastomers.^5,6^ These supersoft materials typically show moduli in the order of 100 Pa, orders of magnitude softer than conventional elastomers or gels. The systematic use of chemistry and/or architecture to access versatile properties for multiple adhesive applications is a significant innovation.

**Figure 2:**
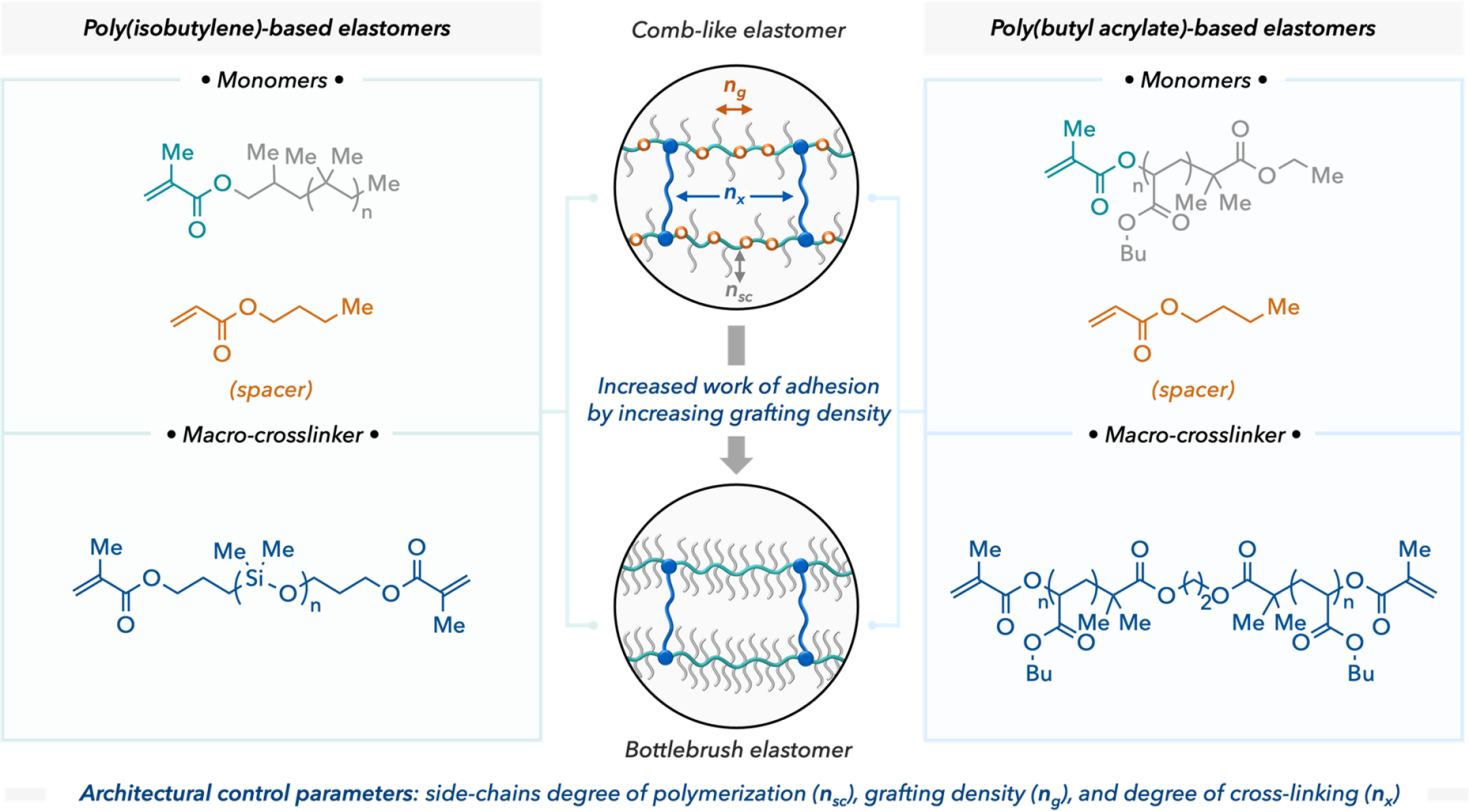
Architectural or chemical properties of the polymer brushes can control the adhesion behavior of the pressure-sensitive adhesives.

The authors show two distinct types of chemistry in their design—poly(isobutylene) (PIB) brushes and poly(butyl acrylate) (PBA) brushes (Figure 2). By varying the cross-link density, length of side chains, and frequency or density of grafted side chains (combs to bottlebrushes), they observed an increase in adhesion with decreasing the cross-link density or increasing the grafting density. In comparing the two types of chemistry, the authors show that identical softness and elastic behavior can be achieved by specific architecture but result in variable adhesion and debonding properties. Additionally, the authors show the tunability of their design (single chemistry) across multiple types of adhesive properties (Chang window),^7^ covering the general application window to high-sheer-rate adhesives. Furthermore, they demonstrate that the design does not require covalent cross-links. Instead, modular adhesion properties can be achieved through microphase separation of disparate polymers, like polystyrene grafts within a poly(isobutylene) brush. Ultimately, the authors can program specific elastic-viscoelastic behavior through multiple variables, including chemistry and distinct architecture.

The structural complexity developed here that is necessary for additive-free, time-dependent adhesive properties is quite remarkable. Indeed, to achieve complex material properties, cooperation between distinct molecular structures across length scales is essential. However, significant challenges remain. Biomedical applications of PSAs are a substantial area of interest.^8^ However, the biocompatibility of bottlebrush-based PSAs and evaluation of their toxicity for biomedical applications remain unknown. In this work, the authors demonstrate the feasibility of this approach for a myriad of applications, with “chemistry-independent control” achieving comparable adhesion properties yet disparate mechanical properties (and vice versa), all tunable through architecture. This generalizable platform signifies opportunities to address function and toxicity by identifying biocompatible chemistry with an appropriate time scale for destruction.

The complex chemistry for additive-free adhesives is necessary to achieve desirable and versatile properties. But the bottlebrush mixed-material architecture and complexity come at a cost. One of the most significant challenges in polymer science is recycling mixed polymer waste, like multilayer packaging, or in this instance, in mixed material adhesives.^9^ Integration of sustainability in designing these polymer adhesives remains a challenge.^10^ More work is necessary to make PSAs recyclable and sustainable. Investigations into selective degradation and/or repurposing of the polymer networks or demonstrating multiple reuses of these adhesives will be critical. Nevertheless, the additive-free architectural design principles and versatility concerning chemistry shown here are a significant step forward to imbuing potential circularity in future materials.
